# Georeferenced data of Christian mission stations, Ghana (1752–1932)

**DOI:** 10.1016/j.dib.2021.107445

**Published:** 2021-10-02

**Authors:** Felix Meier zu Selhausen, Alexander Moradi, Remi Jedwab

**Affiliations:** a Rural and Environmental History, Wageningen University and Research, the Netherlands; b Department of Economics, University of Bozen-Bolzano, Italy; c Department of Economics, George Washington University, USA

**Keywords:** Religion, Christianization, Missionaries, Gold Coast, Ghana, Africa

## Abstract

The data describes Christian mission stations established in Ghana 1752–1932. Data is reported at an annual basis. For all 2,144 mission stations, the data includes station name, denomination, circuity, longitude, latitude, year of entry, exit, whether the station is a main or out-station, and whether it had a school attached. For sub-periods the data also includes information on the number of church members, attendance and seat capacity.

The data was mainly sourced from ecclesiastical returns provided by the mission societies and published in the British Blue Books of the Gold Coast 1844–1932. The source represents a comprehensive census of missions. Various other sources were consulted to extend the data base to Ghana's first mission (1752), to include missions from German Togoland incorporated into Ghana after World War I, and to account for years, for which no Blue Books have survived. Mission stations were then georeferenced based on the place name where the mission is located. Coordinates were retrieved from NGA place name gazetteer as well as other sources.

The data can be used to study patterns in and effects of Christianization in Ghana. The geographic coordinates of the mission stations allow researchers to flexibly link the data to other spatio-temporal databases.

The data has been used in: Jedwab, R., F. Meier zu Selhausen and A. Moradi (2021). Christianization without economic development: Evidence from missions in Ghana.'' Journal of Economic Behavior & Organization **190**: 573–596.

## Specifications Table

**Table:** 

Subject	Social Science
Specific subject area	Economics of Religion, Christianity, Christian Missions, Ghana
Type of data	Table Shapefiles
How data were acquired	Sources were entered into Excel
Data format	Raw Processed Codes (Stata)
Parameters for data collection	All mission stations that existed 1752-1932 within Ghana's boundaries of 2020.
Description of data collection	The data were digitised from archival records.
Data source location	Primary data sources: • Ecclesiastical Returns that missionary societies submitted to the colonial administration on an annual basis and that were published in the Blue Books of the Gold Coast 1844-1932 [1]. • Minutes of the Wesleyan Methodist Church Conferences [2].
Data accessibility	Meier zu Selhausen, Felix; Moradi, Alexander; Jedwab, Remi (2021), “Georeferenced Data of Christian Mission Stations, Ghana (1752-1932)”, Mendeley Data, V1, https://doi.org/10.17632/5r256b72dd.1
Related research article	Jedwab, R., F. Meier zu Selhausen and A. Moradi (2021). ``Christianization without economic development: Evidence from missions in Ghana.'' Journal of Economic Behavior & Organization **190**: 573-596. DOI: https://doi.org/10.1016/j.jebo.2021.07.015

## Value of the Data

• The data represents an annual, complete panel of mission stations in one African country. Studies using these data can address concerns that frequently arise from (selective) misreporting by other sources (e.g. mission atlases).
• The data describes the type of mission, whether main or out-station, with/without mission school and also record temporary entries and exits.
• The data is useful to describe patterns and analyze causes and effects of Christianization in Ghana. It also allows to analyze the full heterogeneity of mission stations that existed.
• The geographic coordinates of missions can be flexibly used to add other spatial data.

## 1 Data Description

### 1.1 Raw data

• <church locations.xls>. This is the raw data entered from the ecclesiastical returns of the Blue Books. The data includes geographic coordinates (“lat” and “lon”), mission society (“Church”), whether main or out-station (“mainstation”), the name of the mission (“place” and alternative spellings of the name (“Diff spelling”), years of existence (“yr1828”, … yr1932_33), name of priest residing at that station “Priest 1857, … “Priest 1870”), church members (“Members 1846”, … “Members 1881”), seat capacity (“Seat Capacity 1861”, …, “Seat Capacity 1899”) and circuity (“Circuity 1857”, … “Circuity 1932”).
• <Togo_church_locations.xls>. We entered the mission data of German Togoland in this file. The data includes geographic coordinates (“lat” and “lon”), mission society (“Church”), whether main or out-station (“type”), the name of the mission (“place” and alternative spellings of the name (“Diff spelling”), years of existence (“togo_yr1847”, … togo_yr1936) and whether the mission was on the territory of the British or French administered part of Togoland (“togo_coloniserpost1918”).
• <school locations.xls>. This file contains the raw data on mission schools from the Education section of the Blue Books.
• <priests.xls>. This data includes background information of the priests stationed in Ghana including the denomination (“church”), duration of residence (“min_year” and “max_year”), and year of death if died in Ghana (“death_yr”).
• <mission_city_coordinates.xls>. This file contains coordinates as well as grid cell identifiers
• <Wesleyan1847_56>. This files contains the data entered from the Minutes of the Wesleyan Methodist Church Conferences.

### 1.2 Do files

The raw data was processed using Stata 16. To replicate the processed data, open the <master_dofile> in Stata, change the working directory, and run the <master_dofile> in Stata. There are sub do files that broke down the coding by topic. All do files detail input files and how input files are processed to output files. Notes are added within the do files.

### 1.3 Processed data

The raw data was cleaned and processed using Stata. The do-files are included in a separate folder. Every step is described in detail there. The output files can be generated running <master_dofile.do>. There are two main output files.

1. <church_locations_panel>. The file contains the panel of missions (“mission”) starting from the year of foundation (yr_startup) to the year of exit (“”) and whether in operation in the respective years (“exist”).. It includes information of the denomination (“church”), whether main or outstation (“mainstation”), school (“school9”), priests known to be stationed there (“priest”).
2. <church_grid.dta>. It contains the same information as <church_locations_panel>. However the unit of observation is a grid cell of 0.1 × 0.1 resolution. The shapefile of the grid can be found in the GIS folder.

### 1.4 GIS data

We added polygon shapefiles of the Muslim and Protestant spheres as well as the 1914 German Togoland border in the GIS folder. All projections are UTM 30N. Finally, we added the grid raster of Ghana (0.1 × 0.1 degrees) on which <church_grid.dta> is based.

## 2 Materials and Methods

Our primary source of mission church data are the Ecclesiastical Returns that missionary societies submitted to the British colonial administration on an annual basis and that were published in the Blue Books of the Gold Coast 1844–1932 [1]. Hence, the data refers to “officially” recognized mission stations.

For certain years ecclesiastical returns are unavailable and we used secondary sources to reconstruct the data.

• 1751–1843: The Blue Books of the Gold Coast start in 1844. The early beginnings of missionary work in Ghana are particularly well-documented and we reconstructed the period 1751–1843 from a variety of secondary sources [3], [4], [5].
• 1862–66: Blue Books were not available in The National Archives (Kew, London). This was a time of a rather stable environment for missionary work. Most of the mission stations in 1861 also existed in 1867. We consulted the Minutes of the Wesleyan Methodist Church Conferences [2], Schlatter [4] and Schreiber [6] for the Methodist, Basel and Bremen Mission, respectively, to confirm dates of new openings and closures.
• 1873–74: During the Third Anglo-Ashanti war 1873–74 many mission stations were abandoned and destroyed. We reconstructed the history of each mission station using a variety of sources [4,7].
• 1917–1919: The Blue Books did not publish ecclesiastical returns. It was impossible to reconstruct the history of each mission station from secondary sources. In 1916, the number of mission stations already exceeded 700. We therefore interpolated assuming that mission stations that existed in 1916 and 1920 also existed 1917–1919. The number of Methodist mission stations stagnated with 311 and 322 in 1916 and 1920, respectively. The assumption seems therefore unproblematic in this case. As for the Basel and Bremen Mission, German and Swiss priests and missionaries were interned during WWI and deported when the war came to an end. The Scottish Mission then took over their mission stations in 1920. We observe a fall in the number of mission stations between 1914 and 1915, from 302 to 215. However, we believe that most of these churches operated 1917–1919, even if under difficult conditions, under the supervision of African personnel. The number of Catholic missions, in contrast, increased from 154 to 256 in 1916 and 1920, respectively. In this case, we ignore the expansion underestimating the number of Catholic missions in 1917–1919.

*Incomplete Ecclesiastical Returns***.** For some years, the Blue Books report main stations or summary statistics but do not report the name of outstations (Basel Mission: 1882, 1885–1887, 1891–1896; Methodist Mission: 1847–56, 1880–1887, 1900–1903). We reconstructed the missing information following three simple rules.

• Firstly, we set those mission stations to exist that according to the Blue Books existed in the year before and after the gap in reporting. For example, the Methodist mission of Komenda was listed in the ecclesiastical returns for the year 1899 and 1904, but not for 1900–1903, as it was an outstation of Elmina. It is likely that Komenda continued to exist, particularly because the period 1900–3 was a time of expansion.
• Secondly, if any mission school was reported in the Blue Books, we assume that the corresponding mission station was also in operation. This assumption is unproblematic, because this is what we overwhelmingly observe: we only found 256 location-years where a school was reported without a mission station out of a total of 6,342 location-years where both school and mission were reported.
• Thirdly, we complemented the Blue Book data with information reported in the Minutes of the Methodist Conferences [2]. We did not add more church locations from this source, but rather restricted the coding to those mission stations that were reported in the Blue Books at least once.

*Obsolete Ecclesiastical Returns***.** We checked whether mission societies updated their returns on a yearly basis and found that this did apparently not occur for the Basel Mission 1911–1913 and the Methodist Mission for the years 1911–12, 1913–14, 1923–24, 1925–27 and 1930–31. We did not attempt to rectify this. In the case of the Basel Mission, 1911–1913 was a time of stagnation, hence measurement error will be small. In the Methodist case, it was a time of expansion, hence the years 1912, 1914, 1924, 1926, 1927 and 1931 may suffer from underreporting.

*Missing Mission Societies*. The Catholic White Fathers in the Northern Territories started reporting only in 1930. We reconstructed their mission locations 1906–1929 using detailed qualitative evidence provided in [7] and [8].

*British Togoland.* German Togoland was occupied by British forces in 1914 and the Western part came under British administration in 1922. British Togoland were only included in the Gold Coast Blue Books from 1920 onwards. We reconstructed the mission stations located in later British Togoland from a wide range of German primary sources. For the years 1890, 1893–1896, 1899–1904, 1918 we used information from the “Deutsches Kolonialblatt” and the “Deutsches Kolonial-Handbuch” [9,10]. For the year 1911, we used a map that showed the location of Bremen mission stations [11]. We assumed that all those mission stations also existed 1912–14. We complemented the remaining years using the information of when mission stations were established from Schreiber [6]. We assumed that mission stations existed unless other sources pointed to the contrary.

*Data Quality Checks.* We assessed data quality by comparing our data with (scattered) information in Bartels [3]; Debrunner [12] and the Encyclopedia of Missions [13] for the period 1840–1900. We were able to match 38 mission stations. The sources largely agree. The difference in the start-up year averages 3 years, which means that churches show up earlier in the Blue Books (the standard deviation being 10.7). We also compared the Blue Book data with stations recorded in the Minutes of the Wesleyan Methodist Conference in 1846, 1857, 1867 and 1879 for which the Ecclesiastical Returns in the Blue Books are complete. We found 104 agreements and 95 deviations, 79 of which are due to the Blue Books listing stations that the Minutes did not list and this is mostly due to the year 1879 when the Minutes stopped comprehensive reporting of out-stations. From the 18 stations that the Minutes reported (and the Blue Books did not), 9 were classified as “vacant”, “agent wanted”. This points to the Blue Books as a source of mission stations where clerical services were actually offered rather than planned to be offered. For a remaining 8 stations, the sources diverged in the start-up-year. Only one place was never listed in the Blue Books (Heginewah - which incidentally might rather be a misspelled place name).

*Latitude and longitude for mission station data:* We georeferenced the location of the churches using National Gespatial-Intelligence Agency [14], a map indexing localities in the 1901 Census [15] and map drawings of missionaries. Overall, we could identify locations 2,096 of the 2,144 church. The remainders were approximated by 0.1 × 0.1 cell centroids.

*Protestant Spheres of Influence:* In 1847, the Methodist and Basel mission agreed on an alliance partitioning the territory into spheres of influence. We digitized the spheres of influence from a map in the Basel mission archives showing the mission fields [16].

*Muslim Sphere of Influence:* We recreated the geospatial location of Muslim sphere of influence using a map based on detailed investigation of the sphere by the Basel missionaries’ (Dr. Rudolf Fisch, Josenhans, Groh) extensive expedition to the Northern Territories in 1910 [17].

## Ethics Statement

The work did not involve the use of human subjects, animal experiments or data collected from social media platforms.

## CRediT authorship contribution statement

**Felix Meier zu Selhausen:** Conceptualization, Investigation, Data curation, Writing – review & editing. **Alexander Moradi:** Conceptualization, Software, Validation, Data curation, Writing – original draft. **Remi Jedwab:** Conceptualization, Formal analysis.

## Declaration of Competing Interest

The authors declare that they have no known competing financial interests or personal relationships which have or could be perceived to have influenced the work reported in this article.

## Appendix Supplementary materials

Image, application 1
